# Curiosities of Cancer: Two Cases of Extraordinary Duration

**Published:** 1913-03-22

**Authors:** 


### CURIOSITIES OF CANCER.

### Two Cases of Extraordinary Duration.

In the literature of cancer all kinds of freakish and curious cases are on record; to these two cases of very unusual prolongation have just been added—one in Australia, the other in Scotland. The first of the pair was that of an old woman, who died recently in Melbourne, at the age of almost ninety-four years, after harbouring a cancer of the breast for forty-seven years! For the last twenty years she had been under the care of one of the medical men who report the case, and before that under two others, now deceased. It appears that forty-seven years before her death she noticed a hard lump in her breast, beginning near the nipple. For ten years it grew very slowly, never reaching any great size, and remaining superficial. In other words, it was a scirrhus cancer of even slower growth than that especially slow-maturing tumour often is. For the next thirty-five years the lump remained quiescent; what it gained by increase of cellular structures it lost by the scarring which forms a part of the scirrhous process. During all this time the woman enjoyed fairly good health. The glands were not affected. Two and a half years before death, without any apparent reason, the growth began to grow rapidly. It spread through the breast, invaded the axillary glands, and then those of the neck and mediastinum, caused brachial paralysis, dysphagia, and eventually death. From the beginning there was dense adhesion to the skin and underlying structures, which seems good proof that the growth was all the time malignant, not merely a case of malignancy supervening in an innocent tumour.

The second case is reported in the *Edinburgh Medical Journal* by Mr. Grieg, of Dundee. At the age of forty a woman was struck on the left breast by the horn of a cow. Eighteen months later she noticed a small hard lump in the breast: the lump was removed by the late Professor Annandale. Eight years after that the growth recurred, and the same surgeon removed the entire breast. After this operation the patient remained well for seven years, after which another recurrence took place and another operation was performed. A year later a further recurrence was dealt with by another surgeon, after which there was an interval of five years before the growth returned, or at least before it became obvious. This time the patient did not submit herself to surgery but waited four years, during which interval the mass grew considerably in size and also became fixed to the deeper tissues.

At the time of the fifth operation the tumour was exceedingly large, for it reached as high as the clavicle and as low as the lower border of the pectoralis major. It was exceedingly soft, and offered all the appearances of a sarcoma. When removed it was found to consist of two parts, each soft, lobulated, and encapsuled. The original growth, it may be mentioned, was hard. At the same time as the large growth was removed the axillary glands were cleared out. After this operation the patient was well for five months, but then began to complain of pain in the shoulder region. Presently a mass appeared above the clavicle; and this bone, the coracoid process of the scapula, and a mass of growth from under the pectoral, were all removed together.

This time more than a year of good health was obtained, but eventually the growth reappeared. Even then its progress was but slow, and more than three years elapsed before the patient finally died at the age of seventy-one years. After death the growth was found to have penetrated into the thorax, and secondary growths were present in several vertebræ. Microscopic sections showed that they were not sarcoma but alveolar carcinoma.

The two cases, published by chance almost at the same date in regions so distant from one another, serve to remind us of the extremely variable and apparently fortuitous course which malignant disease may run. Both cases are, be it understood, highly exceptional; but they do show that while there is life there is hope, even in advanced cases which seem to be inoperable. If certain individuals, with or without the aid of surgical operation, can withstand the attacks of malignant disease, as in these two cases, for forty-seven and thirty years respectively, in the future research may enable us to confer similar resistance upon every sufferer.

